# Foxes fertilize the subarctic forest and modify vegetation through denning

**DOI:** 10.1038/s41598-021-82742-y

**Published:** 2021-02-04

**Authors:** Jessica A. Lang, James D. Roth, John H. Markham

**Affiliations:** Department of Biological Sciences, University of Manitoba, 212B Biological Sciences Building, Winnipeg, MB R3T 2N2 Canada

**Keywords:** Biodiversity, Boreal ecology, Community ecology, Forest ecology

## Abstract

Ecosystem engineers modify habitats through processes other than trophic interactions, such as by regulating soil nutrients, and can influence resource availability and quality for other organisms. Predator-mediated elemental cycling may be especially important in determining plant diversity and growth in ecosystems where soil fertility and primary productivity are low. Red foxes (*Vulpes vulpes* L.), top predators in the Subarctic, could engineer local ecosystems through denning, which could create biogeochemical hotspots of nutrients due to continual input of feces, urine and prey remains. We examined soil and vegetation characteristics on red fox dens and paired control sites in woodland habitats near the Arctic treeline in Manitoba, Canada. The organic soil layer on den sites had 81% more inorganic nitrogen and 250% more extractable phosphorus than in control areas. Denning also increased soil respiration and pH in the organic layer, suggesting improved soil quality and nutrient availability for plants. By enriching nutrients and disturbing soils through digging, den sites had a higher plant species ß-diversity and a greater cover of erect woody shrubs (*Salix* spp.), grasses (*Leymus mollis* (Trinius) Pilger) and weedy ephemerals compared to control sites, resulting in a regional increase in plant species richness. Our research highlights the importance of considering impacts of predators other than through their consumption of prey, and provides insight into the role of red foxes in modifying plant diversity and productivity in the Subarctic.

## Introduction

Apex predators can have profound impacts on ecosystem function and are primarily recognized for their roles in controlling lower trophic levels. For example, by reducing herbivore densities and grazing impacts, predators can indirectly modify vegetation abundance^1–4^. Predators can also influence plant growth and composition through non-trophic interactions, such as by caching prey, mediating seed dispersal, and altering soil properties and other microhabitat conditions^5–9^.

Nutrient deposition by predators through feces, urine, and prey remains can influence the spatial distribution, diversity and growth of plants in many ecosystems^8,10^. For example, brown bears (*Ursos arctos* L.) facilitate the transport of nitrogen by consuming salmon and subsequently excreting urine and feces^11^, thereby increasing nitrogen content in white spruce (*Picea glauca* (Moench) Voss) needles. Similarly, scent-marking by river otters (*Lontra canadensis* Schreber) increases nitrogen content in grasses and mosses by concentrating marine-derived nutrients at coastal latrine sites^12^. This alteration of nutrient availability by predators can be considered a form of ecosystem engineering^13^. Integrating non-consumptive species interactions, such as predator-driven elemental cycling, into the study of food webs is essential to understand community structure and function of ecological networks^14^.

Red foxes (*Vulpes vulpes* L.) are typically known for their impacts on small mammal and waterfowl populations through predation, but they could also influence vegetation through other mechanisms, such as by transporting and recycling nutrients. Red foxes typically give birth to 3–6 pups annually, which remain inside dens for several weeks until fully weaned^15^, resulting in potentially high inputs of nitrogen and phosphorus from feces, urine and prey remains on den sites, as well as frequent soil disturbance by excavating burrows. When denning influences the availability, quality and abundance of resources for other organisms, red foxes may be considered ecosystem engineers. Despite red foxes occupying the largest distribution of any terrestrial carnivore globally^15^, only a few studies have examined their impacts on vegetation, including in temperate forests^16^ and grassland ecosystems^17^. Although red fox denning modified vegetation and soil nutrients in both of these ecosystems, their impacts varied considerably, suggesting that the impacts of red foxes on vegetation are context and ecosystem dependent.

Although the harsh climate previously limited red foxes in the Arctic and Subarctic, warmer temperatures in these regions over the last century may have facilitated the range expansion and increasing densities of red foxes in the North^18,19^. Vegetation in both Subarctic and Arctic ecosystems could be particularly sensitive to red fox denning, given that vegetation may also be responding to climate warming, which has resulted in increased plant productivity and shrub growth on the tundra, as well as decreased productivity in many regions of the boreal forest^20–23^. Impacts of denning near the Arctic treeline could therefore have repercussions for ecosystem function in both the boreal forest and the adjacent tundra. Our objective was to quantify the impacts of red fox denning at the northern boreal treeline by examining den soil properties and vegetation.

Red fox dens could serve as biogeochemical hotspots for soil nutrients due to high inputs of organic waste and prey remains. Soil nitrogen and phosphorus, the main nutrients taken up by plants for growth and metabolism, are typically limited in the boreal forest^24,25^. Increased nutrient availability can increase rates of soil microbial activity^26,27^ and could induce a shift in plant species composition and functional traits from stress-tolerant to competitive strategies^28–30^. Additionally, localized soil disturbance created through digging can create available niches for new plants by removing plant biomass and disrupting the soil profile, allowing ephemeral species to colonize. Therefore, we predicted that soils on red fox dens would have higher concentrations of inorganic nitrogen and extractable phosphorus and higher rates of soil respiration, and that dens would therefore host nutrient-demanding plant species and enhance the growth of weedy ephemerals where soil is frequently disturbed, such as around burrows. Examining the role of red foxes as ecosystem engineers can provide insight into the broad ecological importance of predators in shaping the spatial distribution and diversity of resources across the landscape, which can influence a variety of other organisms.

## Materials and methods

**Figure 1:**
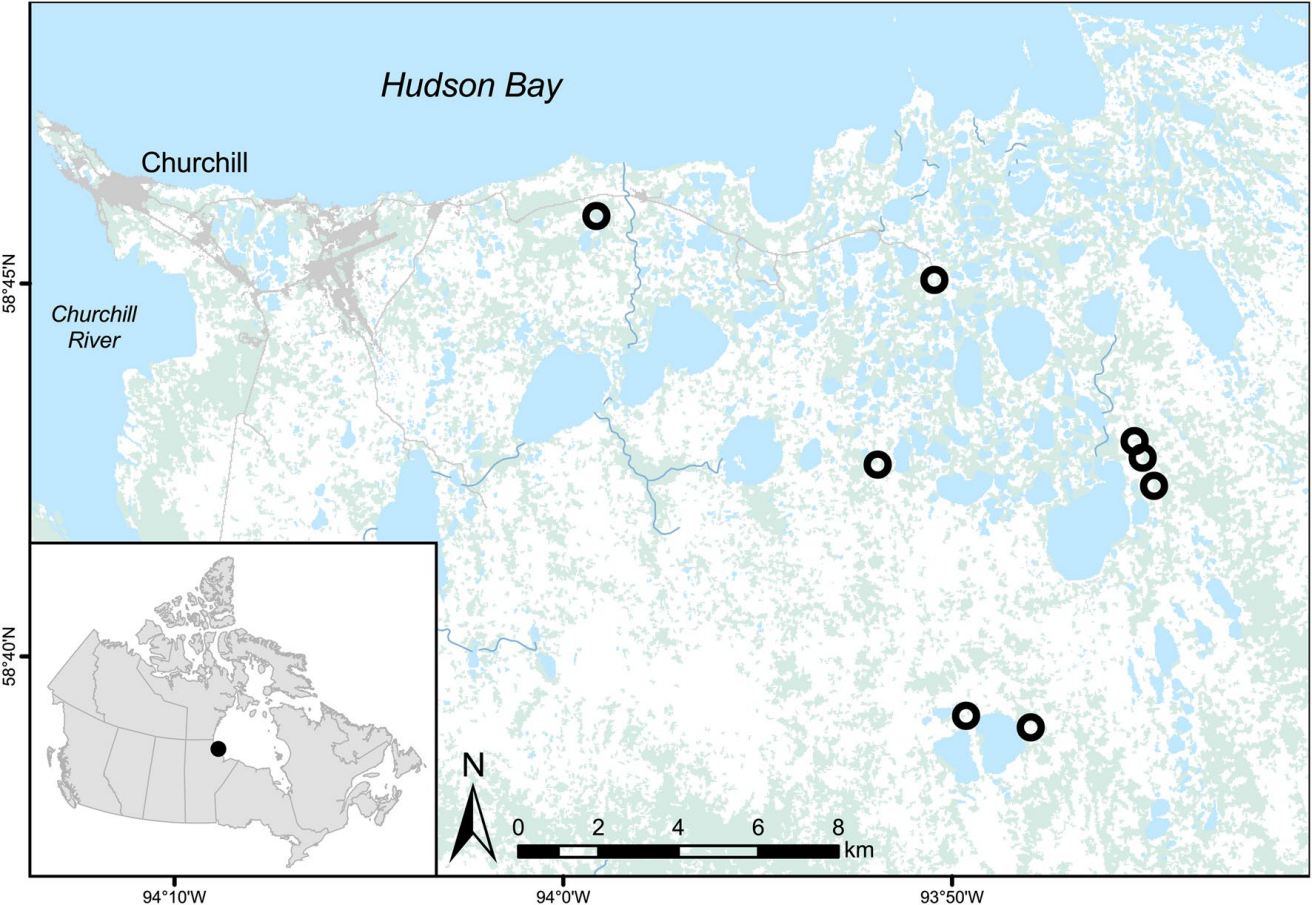
Map of study area near Churchill, Manitoba, Canada (58°46′09″N and 94°10′09″W) indicating red fox dens examined in this study (black circles, n = 8). The map was created using ArcGIS version 10.6.1 (https://desktop.arcgis.com/en/arcmap/)and the 2015 National Land Cover basemap.

Our study was conducted near Churchill, Manitoba (58°46′09″N and 94°10′09″W; Fig. 1), at the transition zone between the northern boreal forest and tundra (Smith et al. 1998). The landscape is dominated by cryosolic soils with dry upland ridges interspersed by bogs and fens. Given the well-drained sandy soils and thick active layer, these ridges provide suitable denning sites for red foxes^31,32^. Vegetation growth in the region is influenced by Hudson Bay, which remains frozen for 7 months each year, and includes erect shrubs (woody shrubs with stems > 15 cm in height), such as willows (*Salix* L. spp.) and dwarf birch (*Betula glanudulosa* Michaud), prostrate shrubs (woody stems < 15 cm in height), such as black crowberry (*Empetrum nigrum* L.) and bog bilberry (*Vaccinium uliginosum* L.), as well as various forbs and lichens^33–35^. White spruce (*Picea glauca* (Moech) Voss) and tamarack (*Larix laricina* K Koch) are the dominant upland tree species along the leading edge of treeline^35–37^. Harvest records suggest both red foxes and Arctic foxes (*Vulpes lagopus* L.) are common predators in the area^31^.

All known fox dens in our study area have been periodically occupied by red foxes each year since at least 1994^31^. Fox dens are assessed annually for activity, using signs of tracks, digging, vocalizations, urine odor and prey remains as indicators (Roth 2003). We used direct sightings and the presence of red fox guard hairs (red, black or gray with striations) around burrows to determine occupancy by red foxes at each den. We examined 8 dens in undisturbed areas of the boreal forest, and excluded another 12 dens found near sites of anthropogenic disturbance (gravel pits or other human excavations) or in forest openings. As each den consists of multiple burrows, we determined the centre of the den as the midpoint along a straight line connecting the two outermost burrows. We designated a paired control site, centred 50 m from the centre of the den, in one of two directions along the ridge to ensure the control site was well outside the area of all burrows and denning activity, while maintaining a similar habitat type, i.e. elevation, slope, soil type^38,39^.

In July 2018, we assessed vegetation at each den and control site using five 1-m^2^ quadrats placed at the site centre and 5 m from the centre in each cardinal direction. The den quadrats were all within the area of the outermost burrows. We visually estimated percent cover for all plant species in each quadrat to the nearest 10% interval. A sample of each species was collected as a voucher specimen to be preserved in the University of Manitoba Vascular Plant Herbarium. While we included seedless plants (Bryophyta, Lycopodiophyta and Marchantiophyta) and lichens in our statistical analyses, most could not be identified to species level. Both *Salix glauca* and *S. athabascensis* (Raup) were found on dens, and their cover was grouped together as they were often indistinguishable in the field.

We sampled soils from both the organic and mineral layers at each quadrat by collecting roughly 250 ml of soil from each corner of every quadrat. Soil samples were air dried until analysis. The depth of the organic layer was recorded for each quadrat, except when the quadrat landed near a burrow, where extensive mixing of soil layers occurred.

We evaluated concentrations of inorganic nitrogen (NO_3_^−^ and NH_4_^+^) and extractable phosphorus (PO_4_^–3^) in each soil sample. Total inorganic nitrogen was determined using the microdiffusion protocol^40^. Phosphorus was extracted using a bray extraction followed by the Murphy Riley assay for phosphate^41^. A subsample of the remaining soil from each quadrat was used to evaluate microbial soil respiration. For each sample, we incubated soil samples in airtight containers after adding 10 ml of deionized water and examined the change in CO_2_ concentrations over a four hour period^42^. We also measured the pH of each soil sample, which was first mixed with deionized water to form a soil slurry.

We used the program R^43^ to conduct all statistical analyses. To compare soil and vegetation characteristics at den and control sites, we used paired t-tests after averaging measurements from the 5 quadrats at each site. To satisfy the test assumptions, we square root transformed inorganic nitrogen and extractable phosphorus concentrations, but no other variables were transformed. We used the Shannon–Wiener diversity index to compare diversity on dens and control sites, as well as the Whittaker β-diversity index followed by a pooled t-test to compare the ratios of total plant species richness among the five quadrats to the average species per quadrat between dens and controls. We used the indicator species analysis^44^ to determine whether plant species were representative of den or control sites, using the labsdv 2.0 package in R^45^. This analysis uses both the frequency and abundance of species to calculate an index of fidelity of species to site types, with a permutation test to calculate the probability of species association with site types. We also compared vegetation functional types (erect shrub, prostrate shrub, forb, grass, sedge, seedless plants and lichens) between den and control sites. Since some functional groups were mostly absent in a site type (the data was zero inflated) we used a Monte Carlo simulation to test for difference in growth forms between sites^46^. This test involved comparing the cover of the functional groups in the two site types to 10 000 randomly generated assignments of the data to the site types.

## Results

**Figure 2:**
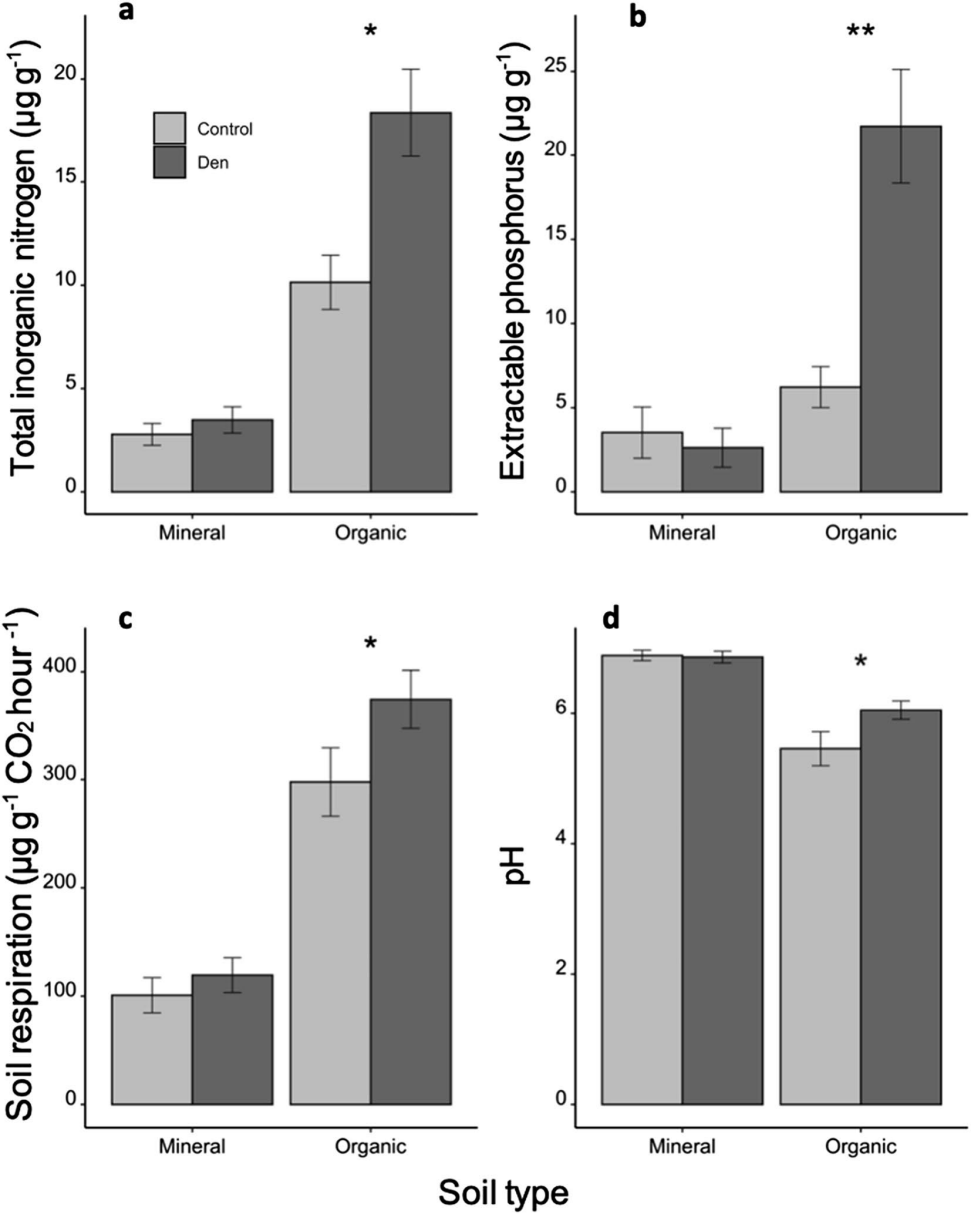
Soil (**a**) inorganic nitrogen, (**b**) extractable phosphorus, (**c**) respiration, and (**d**) pH, in the organic and mineral soil layers from red fox dens and paired control sites (n = 8) near Churchill, MB, Canada, in July 2018. Bars are means and error bars are standard error. *P < 0.05, **P < 0.01.

In the organic soil layer, dens had 81% more total inorganic nitrogen compared to control sites (t_7_ = − 3.288, *P* = 0.013), but in the mineral layer, total inorganic nitrogen did not differ (Fig. 2a; t_7_ = − 1.551, *P* = 0.165). Similarly, the organic layer on dens had 250% more extractable phosphorus compared to control sites (t_7_ = − 5.282, *P* = 0.001), but in the mineral layer, extractable phosphorus did not differ between dens and controls (Fig. 2b; t_7_ = 0.271, *P* = 0.795). Respiration of organic soil was 3 times higher compared to the soil from the mineral layer when incubated under the same conditions on both dens (t_7_ = − 10.386, *P* < 0.001) and controls (Fig. 2c; t_7_ = − 6.548, *P* < 0.001). Dens had higher soil respiration compared to controls in the organic layer (t_7_ = − 3.351, *P* = 0.012), but soil respiration did not differ between dens and controls in the mineral layer (Fig. 2c; t_7_ = − 0.890, *P* = 0.401). The organic layer on dens was more basic (Fig. 2d) compared to control sites (t_7_ = − 2.677, *P* = 0.032). The mineral layer was overall more basic than the organic layer on both dens and controls, but the mineral layer did not differ in pH between dens and controls (Fig. 2d; t_7_ = 1.147, *P* = 0.289). Although the depth of the organic layer did not differ significantly between dens (mean ± SE = 46 ± 4 cm) and controls (31 ± 6 cm; t_7_ = − 1.919, *P* = 0.096), the statistical power was low (0.69), based on the results from a power analysis conducted using the pwr package in R^47^. Increasing our sample size from 8 to 10 paired sites would have achieved significant power (0.80) to detect an effect of denning on organic layer depth, with the same effect size (Cohen’s D = 1.007), suggesting that organic layer was likely deeper on dens.

Species richness (total number of species) was significantly higher on dens (mean ± SE = 17.13 ± 0.789) compared to controls (12.75 ± 0.723; t_7_ = − 5.990, *P* < 0.001). Although vegetation diversity (Shannon diversity index) did not differ between dens (*H*′ = 2.08 ± 0.15) and controls (*H*′ = 1.79 ± 0.079; t_7_ = − 1.644, *P* = 0.1442), diversity between quadrats (Whittaker β-diversity index) was significantly higher on dens (2.01 ± 0.10) than controls (1.74 ± 0.13; t_14_ = 2.179, *P* = 0.047).

**Figure 3:**
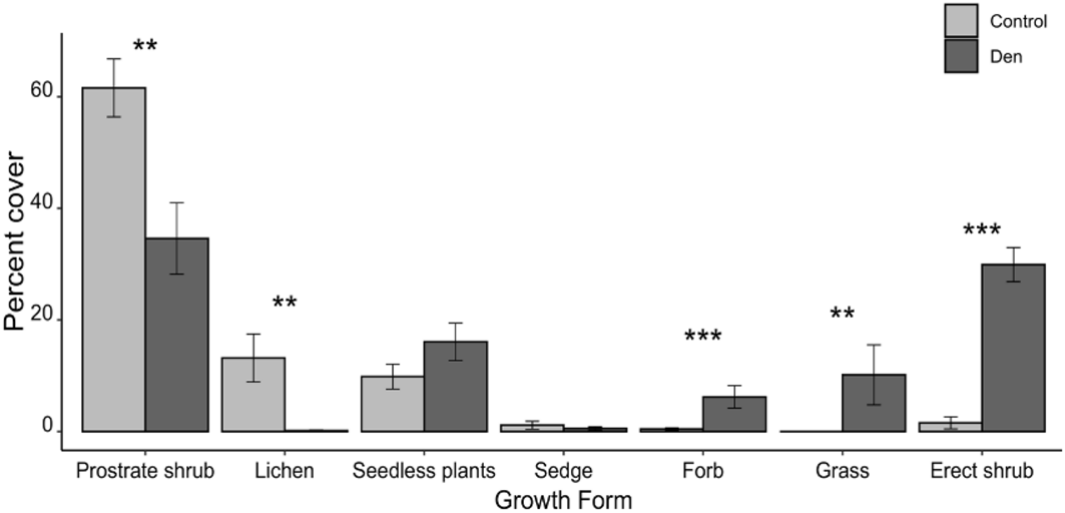
Cover of vegetation growth forms (mean ± SE) from red fox dens and paired control sites near Churchill, MB, Canada, in July 2018. Seedless plants include Bryophyta, Lycopodiophyta and Marchantiophyta. **P < 0.01 and ***P < 0.001 using a Monte Carlo Simulation test.

The indicator species for dens were *Salix glauca* L. and *S. athabascensis*, which were not distinguished in the field, *Chamaenerion angustifolium* L., *Leymus mollis* (Trinius) Pilger and *Pyrola grandiflora* Radius (Table 1, Supplementary Table 1). Indicator species for control sites were *Empetrum nigrum*, *Vaccinium uliginosum*, *Dryas integrifolia* Vahl, and *Cladonia stellaris* L. (Table 1, Supplementary Table 1). Dens had significantly higher cover of erect shrubs (*P* < 0.001), grasses (*P* = 0.007) and forbs (*P* < 0.001) compared to control sites, but lower cover of prostrate shrubs (*P* = 0.006) and lichens (*P* = 0.003) (Fig. 3). Dens and controls did not differ in cover of seedless plants (*P* = 0.145) or sedges (*P* = 0.450) (Fig. 3).

**Table 1:** Relative abundance (percent cover, mean ± SE) and indicator species value of significant indicator plant species on dens and controls (ctrl).

Plant species	Ctrl % cover	Den % cover	Ctrl indicator	Den indicator	*P*
*Dryas integrifolia*	3.0 ± 1.4	0.0 ± 0.0	0.625		0.025
*Empetrum nigrum*	27.9 ± 4.3	10.5 ± 2.8	0.727		0.004
*Vaccinium uliginosum*	14.4 ± 1.8	6.9 ± 2.7	0.678		0.040
*Cladonia stellaris*	12.3 ± 4.3	0.2 ± 0.1	0.988		0.001
*Salix*^a^	0.5 ± 0.5	23.2 ± 3.4		0.979	0.000
*Chamaenerion angustifolium*	< 0.1 ± 0.0	0.7 ± 0.2		0.843	0.004
*Pyrola grandiflora*	< 0.1 ± 0.0	1.7 ± 0.7		0.739	0.011
*Leymus mollis*	0.0 ± 0.0	10.1 ± 5.4		0.625	0.025

^a^Both *Salix glauca* and *Salix athabescensis* were found on dens. Their cover was grouped together as they were often mixed together or indistinguishable in the field.

## Discussion

We found that through the combined effects of nutrient additions and soil disturbance, which was absent on control sites, red foxes enrich soil nutrient concentrations and increase soil respiration, pH and overall species richness at den sites. Red foxes alter soil resource availability, which has been experimentally shown to affect species composition^48^. Given that the organic layer was thick across all sites, which is typical of Subarctic woodlands, the main impacts of denning on soil properties (nutrients, pH, and microbial respiration) were confined to this layer, and the chemistry of the underlying mineral soil was not affected by denning. The organic layer is essential in maintaining soil moisture and nutrients in the boreal forest^27^ and likely plays the major role in storing nutrients from prey remains, feces, and urine. Although typically acidic in the boreal forest^27,49,50^, the den organic layer was more basic. Given the unidirectional nature of burrow excavation, red foxes could increase soil pH by moving mineral soil outwards into the organic layer, without affecting overall pH or nutrients in the mineral layer. Kurek et al.^38^ also attributed the higher pH in soil mounds on red fox and badger (*Meles meles* L.) dens to biopedturbation. Since fox feces are usually slightly basic^51^, the accumulation of feces, as well as frequent urination, may also explain the increase in pH in the den organic layer.

As a reservoir for nutrients and moisture, the thick soil organic layer is essential for supporting higher levels of microbial activity and nutrient mineralization compared to the mineral layer^27,52^. Microbial activity is closely linked to soil pH and is usually reduced in acidic soils^42,50^. Extensive research on the liming effects on boreal organic soils suggest increases in pH can result in long term increases in soil fertility, by increasing base saturation and mineralization rates^53,54^. Higher soil respiration (a proxy for microbial activity) on dens suggests that denning improves substrate conditions and quality for microbial activity, leading to an overall greater uptake of nutrients by plants.

Although enhanced nutrient additions and microbial activity should result in increased litter decomposition^26,55^ and therefore a shallower organic layer, we did not observe any decrease in the organic layer thickness on dens. The abundance of tall shrubs like *Salix* spp. likely increases standing biomass and, therefore, litter accumulation on dens in the absence of strong effects of herbivory^55^, regardless of enhanced microbial activity. Increased litter input on dens compared to controls may therefore negate the differential rate of decomposition in the overall depth of litter and organic material. This observation suggests that long term increased nutrient availability in Subarctic woodlands has the potential to increase carbon storage in the soil organic layer.

Soil pH, nutrient concentrations, microbial activity and disturbance are all known to cumulatively influence plant growth and diversity. As soil nutrient concentrations increase, competition for alternative resources, such as light access or soil moisture, may induce shifts in vegetation diversity^28–30^. Dens were dominated by tall erect shrubs, grasses and forbs, whereas control sites were covered primarily with lichens and prostrate ericaceous shrubs, which are characteristic of Subarctic woodlands. Furthermore, indicator species on control sites all associate with ericoid mycorrhizae, which allows plants to access nutrients in an organic form in areas where soils are nitrogen-deficient and acidic^56,57^. In comparison, den indicator species all associate with arbuscular mycorrhizae, except *P. grandiflora*, and likely utilize the inorganic nitrogen supplied by foxes through excretions and leftover carcasses^58^. Both *L. mollis* and *C. angustifolium,* which were mainly localized around burrows, are also common in frequently disturbed areas^34^, such as gravel pits or alongside roads. Enriched nutrients and nutrient uptake through either microbial or fungal associations, in addition to soil disturbance created by digging, facilitates the growth of a distinct vegetation composition in the area. Additionally, as both nutrient additions and digging are highly localized processes, red foxes can create heterogenous soil conditions at den sites, which may explain the higher β-diversity on dens compared to control sites.

Seeds that make up the plant assemblages on dens may have originated from animal-mediated seed dispersal^16,59,60^. Alternatively, red foxes may facilitate the germination of seeds that are already present in the soil seed bank. In other words, seeds from all of the indicator species found on dens may also be present on control sites, but are unable to establish in the absence of specific soil conditions, such as enriched inorganic nitrogen concentrations or disturbance. Despite creating similar physical or chemical changes in other ecosystems, the response of vegetation to any ecosystem engineering process can vary if the regional species pool differs^61^. For example, although red fox dens on grassland kurgans in Hungary had similarly enriched soil nutrients, denning reduced plant species richness, vegetation cover and litter depth compared to the surrounding area^17^. Unlike at our study area, the strong colonization effects of weedy ephemerals prevented numerous grassland specialist species that were otherwise common in the surrounding landscape from growing on dens, and therefore decreased overall species richness. Another study on red fox dens in a temperate forest in Poland demonstrated high concentrations of phosphorus and plant diversity, but lower nitrogen compared to reference sites^38^. The overall magnitude of impacts of denning and other ecosystem engineering processes can therefore vary in different ecosystems due to differences in regional abiotic conditions^17^. Since impacts of ecosystem engineering are typically more pronounced when they provide a limiting nutrient^62^, regions where soil fertility is high are less likely to be influenced by nutrient additions, but may be more susceptible to soil disturbance from denning.

Like red fox dens in the boreal forest, Arctic fox dens on the tundra, where soil nutrients and species diversity are also low, also have enhanced soil nutrients and distinct vegetation composition^39,58,63^. Notably, in the adjacent tundra in Wapusk National Park, indicator species on Arctic fox dens also included erect *Salix* spp., *L. mollis*, *P.grandiflora*, and *C. Angustifolium*^39,58^. Unlike the tundra studies, where a soil organic layer is almost absent in the upland heath habitat, our results highlight the importance of a thick organic layer in the boreal forest, since differences in soil nutrients, pH and respiration on dens and control sites were not observed in the mineral layer. Red foxes have encroached onto the tundra in recent years (Roth unpublished), where they now occupy dens created by Arctic foxes^31^. Results from our study suggests that red foxes may exert a similar influence on vegetation composition at tundra den sites. Red foxes may therefore contribute to vegetation change on the tundra, similar to Arctic foxes, by contributing to shrub encroachment, which could become amplified under warming conditions^21,64^.

Foxes typically select den sites based on favourable habitat characteristics where prey are abundant^38,65^. Compared to other ecosystems, available sites near Arctic treeline for denning are particularly constrained since red foxes rely on elevated ridges for denning due to the abundance of lowland fens and bogs. Although we cannot confirm that red foxes did not den at sites with already enriched soil and atypical vegetation composition, the similarity of impacts on vegetation documented in other studies, the strong limitation in suitable denning sites and the dependency of foxes in reusing existing dens sites over several decades provides strong evidence that denning has altered vegetation composition regardless of pre-existing vegetation.

Our study shows that red foxes can be conceptualized using an ecosystem engineering framework at the edge of the Arctic, in addition to their roles as predators. Red foxes engineer landscapes on a local scale through denning, and their impacts can have important roles in providing services at the ecosystem level. Carcass sites, which generally have nutrient enriched vegetation, often become foraging sites for herbivores^66^. By altering vegetation, red foxes could also provide ecosystem services to herbivores, like Arctic foxes on the tundra^67^. By denning at the transition zone between the boreal forest and tundra, red foxes may influence resources used by both boreal forest and tundra organisms. Although the impacts of denning occur at a local scale, the lifespan and collective impacts of all red fox dens could have implications for long-term landscape and ecosystem function.

## Supplementary Information

Supplementary Table 1.
