# Supplementary material for: Foxes fertilize the subarctic forest and modify vegetation through denning
Source: Sci Rep. 2021 Feb 4;11:3031. doi: 10.1038/s41598-021-82742-y (PMC7862318; doi:10.1038/s41598-021-82742-y)
Supplement: Supplementary file 1 — Supplementary Table 1. [file 41598_2021_82742_MOESM1_ESM.pdf]

# FOXES FERTILIZE THE SUBARCTIC FOREST AND MODIFY VEGETATION THROUGH DENNING

**Electronic Supplemental Material**

Jessica A. Lang, James D. Roth, and John H. Markham

Department of Biological Sciences, University of Manitoba. 212B Biological Sciences Building, Winnipeg, MB, Canada, R3T 2N2.

Email addresses: jessicaashley.lang@gmail.com; Jim.Roth@umanitoba.ca; John.Markham@umanitoba.ca

*Corresponding author: John.Markham@umanitoba.ca

**Supplementary Table 1.** Relative abundance (percent cover, mean ± SE) and indicator analysis results (indicator values and p-values) for plant species on dens and control sites (ctrl). Significant indicator values are bolded ($p < 0.05$).

| Plant species | Ctrl % cover | Den % cover | Ctrl indicator | Den indicator | p |
|---|---|---|---|---|---|
| **Prostrate shrubs** | | | | | |
| *Andromeda polifolia* | 0.5 ± 0.4 | 0.0 ± 0.0 | 0.250 | 0.000 | 0.462 |
| *Arctostaphylous* sp[a] | 2.2 ± 1.1 | 5.6 ± 2.0 | 0.210 | 0.630 | 0.185 |
| *Dryas integrifolia* | 3.0 ± 1.4 | 0.0 ± 0.0 | **0.625** | 0.000 | **0.025** |
| *Empetrum nigrum* | 27.9 ± 4.3 | 10.5 ± 2.8 | **0.727** | 0.273 | **0.004** |
| *Kalmia procumbens* | 1.4 ± 1.4 | 0.0 ± 0.0 | 0.125 | 0.000 | 1.000 |
| *Rhododendron tomentosum* | 4.6 ± 3.0 | 1.9 ± 1.5 | 0.353 | 0.074 | 0.360 |
| *Rubus arcticus subsp. acaulis* | 0.0 ± 0.0 | 0.8 ± 0.6 | 0.000 | 0.375 | 0.204 |
| *Salix reticulata* | 0.2 ± 0.1 | 1.8 ± 1.3 | 0.041 | 0.556 | 0.269 |
| *Shepherdia canadensis* | 5.5 ± 2.4 | 3.0 ± 2.2 | 0.565 | 0.177 | 0.244 |
| *Vaccinium uliginosum* | 14.4 ± 1.8 | 6.9 ± 2.7 | **0.678** | 0.322 | **0.040** |
| *Vaccinium vitis-idaea* | 2.0 ± 0.4 | 4.1 ± 1.2 | 0.327 | 0.673 | 0.113 |
| **Erect shrubs** | | | | | |
| *Betula glandulosa* | 0.9 ± 0.6 | 4.7 ± 1.8 | 0.062 | 0.627 | 0.063 |
| *Juniperus communis* | 0.1 ± 0.1 | 1.6 ± 1.6 | 0.009 | 0.116 | 1.000 |
| *Salix*[b] | 0.5 ± 0.5 | 23.2 ± 3.4 | 0.003 | **0.979** | **0.000** |
| *Ribes hudsonianum* | 0.0 ± 0.0 | 0.4 ± 0.3 | 0.000 | 0.250 | 0.467 |
| **Forbs** | | | | | |
| *Achillea millefolium* | 0.0 ± 0.0 | 0.3 ± 0.2 | 0.000 | 0.375 | 0.203 |
| *Anemone richardsonii* | 0.0 ± 0.0 | 0.5 ± 0.3 | 0.000 | 0.375 | 0.204 |
| *Bartsia alpina* | < 0.1 ± 0.0 | 0.0 ± 0.0 | 0.125 | 0.000 | 1.000 |
| *Bistorta vivipara* | 0.3 ± 0.3 | 0.0 ± 0.0 | 0.125 | 0.000 | 1.000 |
| *Chamaenerion angustifolium* | < 0.1 ± 0.0 | 0.7 ± 0.2 | 0.005 | **0.843** | **0.004** |
| *Fragaria virginiana* | 0.0 ± 0.0 | 0.7 ± 0.4 | 0.000 | 0.500 | 0.078 |
| *Geocaulon lividum* | 0.1 ± 0.1 | 0.8 ± 0.5 | 0.008 | 0.352 | 0.205 |
| *Moneses uniflora* | 0.0 ± 0.0 | 0.3 ± 0.3 | 0.000 | 0.125 | 1.000 |
| *Mitella nuda* | 0.0 ± 0.0 | 0.2 ± 0.1 | 0.000 | 0.375 | 0.201 |
| *Pedicularis flammea* | < 0.1 ± 0.0 | < 0.1 ± 0.0 | 0.063 | 0.063 | 1.000 |
| *Pyrola grandiflora* | < 0.1 ± 0.0 | 1.7 ± 0.7 | 0.002 | **0.739** | **0.011** |
| *Solidago multiradiata* | 0.0 ± 0.0 | 1.2 ± 1.0 | 0.000 | 0.500 | 0.072 |
| *Stellaria longipes* | < 0.1 ± 0.0 | < 0.1 ± 0.0 | 0.063 | 0.063 | 1.000 |

| Plant species | Ctrl % cover | Den % cover | Ctrl indicator | Den indicator | P |
|---|---|---|---|---|---|
| **Graminoids** | | | | | |
| *Carex concinna* | 0.5 ± 0.5 | 0.0 ± 0.0 | 0.125 | 0.000 | 1.000 |
| *Carex scirpoidea* | 0.6 ± 0.6 | 0.3 ± 0.2 | 0.171 | 0.157 | 1.000 |
| *Carex vaginata* | 0.0 ± 0.0 | 0.3 ± 0.3 | 0.000 | 0.125 | 1.000 |
| *Leymus mollis* | 0.0 ± 0.0 | 10.1 ± 5.4 | 0.000 | **0.625** | **0.025** |
| *Poa arctica* | 0.0 ± 0.0 | 0.1 ± 0.1 | 0.000 | 0.375 | 0.199 |
| **Seedless plants (Bryophyta, Lycopodiophyta and Marchantiophyta)** | | | | | |
| *Dicranum* spp. | 2.0 ± 1.3 | 1.4 ± 1.0 | 0.221 | 0.309 | 0.892 |
| *Lycopodium annotinum* | 0.0 ± 0.0 | 0.3 ± 0.3 | 0.000 | 0.125 | 1.000 |
| *Moss* sp. | < 0.1 ± 0.0 | 2.0 ± 2.0 | 0.002 | 0.123 | 1.000 |
| *Plagiochila* spp. | 0.0 ± 0.0 | < 0.1 ± 0.0 | 0.000 | 0.125 | 1.000 |
| *Pleurozium schreberi* | 7.8 ± 2.3 | 12.3 ± 1.7 | 0.340 | 0.611 | 0.131 |
| **Lichens** | | | | | |
| *Cladonia*[c] *sp* | 0.1 ± 0.1 | 0.0 ± 0.0 | 0.250 | 0.000 | 0.454 |
| *Cladonia bacilliformis* | < 0.1 ± 0.0 | 0.0 ± 0.0 | 0.125 | 0.000 | 1.000 |
| *Cladonia stellaris* | 12.3 ± 4.3 | 0.2 ± 0.1 | **0.988** | 0.003 | **0.001** |
| *Flavocetraria nivalis* | 0.3 ± 0.2 | 0.0 ± 0.0 | 0.500 | 0.000 | 0.076 |
| *Lichen* spp. | 0.5 ± 0.5 | 0.0 ± 0.0 | 0.125 | 0.000 | 1.000 |
| *Peltigera* spp. | 0.0 ± 0.0 | 0.1 ± 0.1 | 0.000 | 0.250 | 0.475 |
| **Trees** | | | | | |
| *Larix laricina* | 1.0 ± 0.5 | 0.0 ± 0.0 | 0.375 | 0.000 | 0.200 |
| *Picea glauca* | 3.3 ± 1.8 | 1.2 ± 0.4 | 0.461 | 0.164 | 0.472 |

[a] Given the absence of berries, we could not distinguish between *Arctostaphylous rubra* and *Arctostaphylous alpina*.

[b] Both *Salix glauca* and *Salix athabescensis* were found on dens. Their cover was grouped together as they were often mixed together or indistinguishable in the field.

[c] *Cladonia fimbriata* and *Cladonia chlorophaea.*
